# Ultimate Secreting Structure

**Published:** 1842-08-27

**Authors:** 


*Ultimate Secreting Structure.*—Mr. Goodsir, in a communication read before the Royal Society of Edinburgh, on the ultimate secreting structure, and on the laws of its function, after referring to the labours of those anatomists who had confirmed Malpighi's doctrine of the follicular nature of gland ducts, alludes to Purkinje's hypothesis of the secreting function of the nucleated corpuscles which line these ducts. He then proceeds to state, that no anatomist had hitherto proved that secretion takes place within the primitive nucleated cell itself, or had pointed out the intimate nature of the changes which go on in a secreting organ during the performance of its function. The ink of the cephalopoda and the purple of janthina and aplysia, bile in an extensive series selected from the principal divisions of the animal kingdoms, urine in the mollusk, milk, &c., are all considered by Mr. Goodsir as examples of secretions detected in the cavities of nucleated cells of various glands and secreting surfaces.

The wall is believed by the author to be the part of the cell engaged in the process of secretion. The cavity contains the secreted substance, and the nucleus is the reproductive organ of the cell. A primitive cell engaged in secretion is denominated by him a primary secreting cell; and each cell of this kind is endowed with its own peculiar property, according to the organ in which it is situated. The discovery of the secreting agency of the primitive cell does not remove the principal mystery in which the function has always been involved; but the general fact that the primitive cell is the ultimate secreting structure, is of great value in physiological science, inasmuch as it connects secretion with growth, as functions repeated by the same laws, and explains one of the greatest difficulties in physiology—viz., why a secretion flows from the free surface of a secreting membrane—the secretion exists only on the free surface of the ripe cells which constitute that surface.

The origin, developement, and disappearance of the primary secreting cell, a subject necessarily involving the description of the various minute arrangements of glands and other secreting organs, come next under consideration. The testicle of the squalus cornubicus and the liver of the careinus mænas are adduced as examples of two orders of glands, denominated by our author vesicular and follicular. The changes which occur in the first order of glands consist in the formation and disappearance of closed vesicles or acini. Each acinus might be first a single cell, the primary or germinal cell; or, secondly, it might consist of two or more cells enclosed in the primary cell, and produced from its nucleus. These enclosed cells are the secondary cells of the acinus, and in their cavities, between their nuclei and cell-walls, the peculiar secretion of the gland is contained. The primary cell, with its included group of cells, each full of secretion, is appended to the extremity of one of the terminal ducts, and consequently does not communicate with that duct, a diaphragm formed by a portion of the primary cell wall stretching across the pedicle. When the secretion in the group of cells is fully elaborated, the diaphragm dissolves or gives way, the cells burst, and the secretion flows along the ducts, the acinus disappearing, and making room for a neighboring acinus, which has in the mean time been advancing in a similar manner. The whole parenchyma of glands of this order is thus, according to Mr. Goodsir, in a constant state of change—of developement, maturity, and atrophy—this series of changes being directly proportional to the profuseness of the secretion.

In the second order of glands, the follicular, the germinal cell or spot is situated at the blind extremity of the follicle, and the secreting cells, as they advance along the follicle, become distended with their peculiar secretion.

From all these facts, Mr. Goodsir infers that secretion is a function of, and takes place within, the nucleated cell; and that growth and secretion are identical—the same process under different circumstances.—*London and Edinburgh Monthly Medical Journal.*

